# The impact of Brazil’s Bolsa Família conditional cash transfer program on children’s health care utilization and health outcomes

**DOI:** 10.1186/1472-698X-14-10

**Published:** 2014-04-01

**Authors:** Amie Shei, Federico Costa, Mitermayer G Reis, Albert I Ko

**Affiliations:** 1 Analysis Group, Inc., 111 Huntington Avenue, 10th Floor, Boston, MA 02199, USA; 2 Fundação Oswaldo Cruz (FIOCRUZ), Centro de Pesquisas Gonçalo Moniz, Rua Waldemar Falcão, 121, Candeal, Salvador, BA 40296-710, Brazil; 3 Department of Epidemiology of Microbial Diseases, Yale School of Public Health, 60 College Street, LEPH Room 319B, P.O. Box 208034, New Haven, CT 06520-8034, USA; 4 Bahia School of Medicine, Federal University of Bahia, Largo do Terreiro de Jesus - Centro Histórico, Salvador, BA 40025-010, Brazil; 5 Bahiana School of Medicine and Public Health, Av. Dom João VI, 274, Brotas, Salvador, BA 40285-001, Brazil

## Abstract

**Background:**

Conditional cash transfer (CCT) programs provide poor families with cash conditional on investments in health and education. Brazil’s Bolsa Família program began in 2003 and is currently the largest CCT program in the world. This community-based study examines the impact of Bolsa Família on child health in a slum community in a large urban center.

**Methods:**

In 2010, detailed household surveys were conducted with randomly selected Bolsa Família beneficiaries and non-beneficiaries in a Brazilian slum community of approximately 14,000 inhabitants in a large urban center. 567 families (with 1,266 children) were interviewed. Propensity score methods were used to control for differences between beneficiary and non-beneficiary children to estimate program impacts on health care utilization and health outcomes.

**Results:**

Bolsa Família has increased the odds of children’s visits to the health post for preventive services. In children under age seven, Bolsa Família was associated with increased odds for growth monitoring (OR = 3.1; 95% CI 1.9-5.1), vaccinations (OR = 2.8; 95% CI 1.4-5.4), and checkups (OR = 1.6; 95% CI 0.98-2.5), and with the number of growth monitoring visits (β = 0.6; p = 0.049) and checkups (β = 0.2; p = 0.068). There were positive spillover effects on older siblings (ages 7-17) no longer required to meet the health conditionalities. Bolsa Família increased their odds for growth monitoring (OR = 2.5; 95% CI 1.3-4.9) and checkups (OR = 1.7; 95% CI 0.9-3.2) and improved psychosocial health (β = 2.6; p = 0.007).

**Conclusions:**

Bolsa Família has improved health care utilization, especially for services related to the health conditionalites, and there were positive spillover effects on older siblings. The findings of this study are promising, but they also suggest that further improvements in health may depend on the quality of health care services provided, the scope of services linked to the health conditionalities, and coordination with other social safety net programs.

## Background

Brazil’s Bolsa Família program is currently the largest conditional cash transfer (CCT) program in the world in terms of coverage and financing. Over the last decade, CCT programs have emerged as a popular social safety net in developing countries and an innovative approach to alleviating poverty. CCT programs aim to reduce poverty in the short-term by providing poor families with cash and improve human capital in the longer-term by encouraging behaviors related to health, nutrition, and education. Poor children are often disadvantaged from the start, as poor parents are less able to invest in their children’s health and education, and poverty continues from one generation to the next. CCT programs attempt to break this inter-generational cycle of poverty. Even when health care services are widely available, poor families are not always able to access them due to a variety of barriers such as fees, transportation costs, or time off from work. Because effective health care is often underutilized, health improvements in developing countries may not reach their full potential [1]. CCT programs are demand-side tools that encourage poor families to utilize existing health care services. They have been widely implemented and are now present in approximately 30 countries [2].

Evidence from other countries suggests that CCT programs have improved the lives of people in poverty. Reported benefits include increased consumption among the poor, decreased poverty, protection from income shocks such as unemployment and catastrophic illness, and increased bargaining power of women [2]. In terms of health, CCT programs have increased the use of preventive health services [3-6] and improved some child and adult health outcomes [3,7,8].

Brazil’s Bolsa Família CCT program was created in 2003. Program eligibility is based on per capita household income, and the benefit amounts vary from R$ 22-200 (US$ 11-98) for the study period, depending on family composition and income. Monthly payments are made preferentially to women and are directly credited to beneficiaries’ electronic benefit cards conditional on compliance with health and education conditionalities. Children under the age of seven years are expected to comply with Brazil’s childhood immunization schedule [9] and to make growth monitoring visits twice a year. Children between the ages of 6-17 years are expected to enroll in school and maintain minimum daily school attendance of 85% (75% for ages 16-17). Schools and health centers are responsible for reporting compliance.

The program currently covers over 13 million families [10], approximately one quarter of all Brazilian households. Over its eight years of existence, it has spent R$ 76 billion [11]. Despite being the largest CCT program, evidence on program impacts is limited. Unlike Mexico’s Oportunidades and Ecuador’s Bono de Desarollo Humano CCT programs, which implemented randomized experiments to evaluate impacts, Bolsa Família had a goal of rapid and universal coverage of the poor [12], and no evaluation strategy was in place when the program was implemented. As a result, much less is known about Bolsa Família’s effect on consumption, poverty, health, nutrition, and education [2].

Several studies have examined Bolsa Família’s impacts on equity [13,14], decision-making [15], food security and nutrition [16-21], and health and education services [22]. Among these, many have methodological weaknesses such as the lack of comparison groups, and the outcomes examined were limited to vaccinations and nutritional status. However, more recent investigations, which used ecological study designs, have reported an important positive effect of Bolsa Família on child mortality [23] and infant mortality [24]. For example, Bolsa Família was found to have contributed to a significant reduction in mortality in children younger than five years of age; this effect was strongest for deaths attributable to poverty-related causes, such as diarrhea and malnutrition [23]. Herein, we report the findings of a population-based study which was performed in a slum community in the city of Salvador, Brazil and explores the mechanisms at work behind the positive outcomes of Bolsa Família by evaluating its impacts on children’s utilization of health care services, illness rates, and overall physical and psychosocial health.

## Methods

### Data collection

Between June and September, 2010, household surveys were conducted with beneficiary and non-beneficiary families in an urban slum community (*favela*) in the city of Salvador, which is situated in Northeast Brazil. The northeast region receives a disproportionately large share of Bolsa Família funds due to its high prevalence of poverty.

This study built upon a long-term prospective cohort investigation of slum residents which was initiated in 2003 to study the transmission dynamics of leptospirosis [25,26] and other urban-slum-associated infectious diseases [27]. The 2008 census of the project site (comprised of approximately 14,000 inhabitants residing in an area of 0.52 km^2^) was used as the sampling frame for this study, and a random number table was used to select a sample of 3,000 households. Selected households were visited by trained interviewers and invited to participate in the study if they met the following inclusion criteria: had at least one child under the age of seven, had a monthly per capita household income of R$ 250 or less, and did not include multiple families. The study’s income cut-off was intentionally set above the program’s eligibility cut-off because exploratory research indicated that many families with monthly per capita household income above the formal cut-off were participating in the program. (This may be due to imperfect program targeting, fluctuations in income and family composition, or the fact that registration information is only updated every two years).

The questionnaire was administered to the mother or female head of household and included questions about the household and all household residents. Informed consent was obtained from all respondents, and the questionnaire was administered in Portuguese. Households were excluded from the study if the house was abandoned, under construction, or in ruins; the respondent refused participation; the respondent was not home on three separate visits (including at least one weekend visit); or the household could not be located after three separate attempts. The response rate was 88% (Response Rate 3 definition [28]). A diagram of the study design and inclusion and exclusion criteria is presented in Additional file 1: Figure S1.

For each child, data about program status, outcomes (health care utilization and health status), and covariates were collected. Children were classified as beneficiaries if the respondent reported that the household currently received Bolsa Família. Each child’s health care utilization history for 2009 was obtained for different facilities: the health post, urgent care center, and hospital. Health post visits were further separated by purpose: growth monitoring, vaccinations, routine checkups or well visits, and sick visits. The first two purposes are directly targeted by the health conditionalities.

Illness was measured by occurrence of diarrhea in the last three months and last two weeks, fever in the last two weeks, and cough in the last two weeks. Health status was measured using questions based on the QualityMetric Incorporated SF-10™ Health Status questionnaire for assessing the physical and psychosocial functioning of children ages five and older [29]. While the SF-10 questionnaire has been translated and validated for many countries, a version for use in Brazil (Portuguese) was not available. Therefore, questions were adapted from the Portugal (Portuguese) version. The physical health and psychosocial health summary measures are calculated as recommended by the SF-10™ User’s Guide [29]. Other observed covariates included characteristics about the child (e.g., age, sex, education), mother (e.g., age, education, work status), and household (e.g., participation in government programs including Bolsa Família, ownership of home, sanitation).

Participation in the study was voluntary, and participants were not offered any compensation for their participation. Institutional review board approval was obtained from Harvard University, the Research Ethics Committee (Comitê de Ética em Pesquisa) at the Oswaldo Cruz Foundation–Brazilian Ministry of Health, and the National Research Ethics Committee (Comissão Nacional de Ética em Pesquisa) in Brazil.

### Statistical analysis

Propensity score adjustment was used to remove bias associated with differences in the distributions of observed covariates in beneficiary and non-beneficiary groups [30,31]. A previous CCT impact evaluation using a propensity score method yielded results similar to those with an experimental design [32]. The propensity score is the propensity (probability) of being a Bolsa Família beneficiary given observed covariates for the child, mother, and household (Table 1). Propensity score estimation was conducted using logistic regression models for different age subgroups (Table 2) determined a priori based on the ages targeted by the health conditionalities (children under 7) and the health status questions (children 5 and older [29]).

**Table 1:** Household and individual characteristics

	**Before weighting**			**After weighting**		
	**In BF HH**	**In non-BF HH**	**p value**	**In BF HH**	**In non-BF HH**	**p value**
	**(n = 776)**	**(n = 343)**				
*Household’s characteristics*						
Ownership of home	85.18	87.17	0.572	86.28	86.28	1.0
Title to home	8.76	18.08	0.009***	14.51	14.51	1.0
Functioning water meter	6.31	10.79	0.095*	8.44	8.44	1.0
Functioning light meter	52.96	61.22	0.115	59.27	59.27	1.0
Closed sewer	54.51	69.10	0.004**	64.57	64.57	1.0
Monthly per capita household income	86.52 (64.80)	102.64 (70.82)	0.017**	102.30 (4.84)	102.30 (6.13)	1.0
Receipt of another government benefit	23.97	36.44	0.011**	29.74	29.74	1.0
# household members	5.54 (2.21)	4.62 (1.61)	0.000***	4.81 (0.14)	4.81 (0.19)	1.0
At least one child between the age of 7 to 17 years	82.09	59.48	0.000***	68.65	68.65	1.0
At least one member self-employed	53.74	45.19	0.101	50.31	50.31	1.0
At least one member informally employed	23.32	13.12	0.008***	15.60	15.60	1.0
At least one member formally employed	34.28	48.10	0.007***	45.97	45.97	1.0
*Mother’s characteristics*						
Mother’s possession of CPF	97.16	93.59	0.114	96.60	96.60	1.0
Mother’s race – black	60.31	51.31	0.084*	55.18	55.18	1.0
Mother’s race – mixed	34.41	40.23	0.254	36.09	36.09	1.0
Mother’s age	35.54 (8.21)	33.39 (10.89)	0.023**	33.63 (0.61)	33.63 (0.79)	1.0
Mother’s age, squared	1330.58 (640.81)	1232.93 (898.47)	0.199	1211.96 (47.60)	1211.96 (58.44)	1.0
Mother currently in school	9.54	8.16	0.601	8.74	8.74	1.0
Mother is literate	70.23	76.38	0.195	78.33	78.33	1.0
Mother’s education level education – fundamental school	71.78	57.14	0.003***	64.02	64.02	1.0
Mother’s education level – above fundamental school	22.81	38.78	0.001***	33.16	33.16	1.0
Mother has a religion	84.54	77.26	0.088*	81.20	81.20	1.0
Mother knows a family receiving BF	97.94	88.92	0.000***	95.36	95.36	1.0
Mother is a member in a group	16.24	11.66	0.180	12.91	12.91	1.0
Mother always votes	86.21	80.47	0.120	84.40	84.40	1.0
*Child’s characteristics*						
Child’s possession of ID	40.21	38.78	0.684	37.19	37.19	1.0
Child’s possession of CPF	7.09	11.08	0.070*	8.46	8.46	1.0
Child’s race – black	44.85	38.19	0.137	41.88	41.88	1.0
Child’s race – mixed	47.42	49.85	0.591	48.17	48.17	1.0
Child’s age	8.35 (4.46)	6.96 (4.24)	0.000***	7.25 (0.20)	7.25 (0.27)	1.0
Child’s age, squared	89.55 (85.07)	66.32 (76.60)	0.000***	70.98 (3.69)	70.98 (5.15)	1.0
Child’s gender – female	53 · 74	48.98	0.163	50.11	50.11	1.0

Data are % or mean (SD). Age was calculated as of the date of the interview. Table includes all children born before 2009. p values are for cluster-adjusted t test (continuous variables) or χ2 test (dichotomous variables) tests of independence. *p < 0.10, **p < 0.05, ***p < 0.01.

**Table 2:** Outcomes and Age/Gender subgroups

**Outcomes and measures**	**Age subgroups (and sample sizes)**
Health care utilization (dichotomous and continuous)	• Less than the age of 7 years (n = 630)
Visits to health post – growth monitoring	• Between the age of 7-17 years (n = 489)
Visits to health post – vaccinations	
Visits to health post – checkup	
Visits to health post – sick visit	
Visits to urgent care center	
Visits to hospital	
Illness	• Less than the age of 7 years (n = 723)
Diarrhea in the last 3 months	• Between the age of 7-17 years (n = 517)
Diarrhea in the last 2 weeks	
Fever in the last 2 weeks	
Cough in the last 2 weeks	
Health status	• Between the age of 5-7 years (n = 246)
10 individual items	• Between the age of 7-17 years (n = 517)
Physical health summary measure	
Psychosocial health summary measure	

Age was calculated as of the interview date except in the analysis of health care utilization when age was calculated as of December 31, 2009. Children are at least 3 months old in the sub-group used for the analysis of illness rates. Children less than 5 years old are excluded from the analysis of health status because the health status questions were only applied to children ages 5 and older.

Propensity score weighting was then used to reweight beneficiary and non-beneficiary observations to be representative of the population of interest [30]. Each child was assigned a “population-overlap weight” (propensity of being in the opposite group) [33]. This technique balances characteristics to resemble those among overlapping portions of the treatment and control distributions of observed characteristics, thereby minimizing the variance of the estimates [33]. Additional file 1: Note A1 further describes the propensity score weighting technique. The weights were then used in logistic and linear regression models to estimate the impact of Bolsa Família on dichotomous and continuous outcomes, respectively. All analyses were conducted using Stata version 11 (StataCorp LP, College Station, Texas).

### Role of funding source

The sponsors of the study had no role in the study design, data collection, analysis, and interpretation, or writing of the report. The corresponding author had full access to all the data and takes responsibility for the integrity of the data and accuracy of the data analysis and had final responsibility for the decision to submit for publication.

## Results

Data were collected on 1,266 children: 841 (66%) beneficiary and 425 (34%) non-beneficiary children. Table 1 shows the individual and household characteristics of beneficiary and non-beneficiary children born before 2009 (n = 1,119). The beneficiary and non-beneficiary groups differ significantly on several covariates. As an example, beneficiary children are older and more likely to live in larger and poorer households and have less educated mothers, among other observed differences. After propensity score adjustment, however, all observed characteristics used in the propensity score estimation were balanced between the two groups (Table 1). Table 3 summarizes the outcome variables by sub-groups.

**Table 3:** Outcome variables, by sub-groups

	**Children < 7 years**	**Children 7-17 years**
**Health care utilization (dichotomous)**		
Any health post visit – growth monitoring	440 (70.74)	203 (42.29)
Any health post visit – vaccination	540 (88.38)	110 (23.01)
Any checkup	380 (60.90)	219 (45.06)
Any health post visit – sick	138 (22.29)	67 (13.73)
Any urgent care center visit	430 (69.13)	219 (44.97)
Any hospital visit	95 (15.20)	31 (6.35)
**Health care utilization (continuous)**		
Health post visits – growth monitoring	2.39 (3.08)	0.97 (1.72)
Health post visits – vaccination	2.20 (1.81)	0.33 (0.74)
Checkups	1.11 (1.38)	0.67 (1.02)
Health post visits – sick	0.50 (1.15)	0.25 (0.82)
Urgent care center visits	1.80 (2.26)	0.88 (1.36)
Hospital visits	0.25 (0.84)	0.11 (0.62)
**Illness**		
Diarrhea in last 3 months	223 (30.84)	81 (15.67)
Diarrhea in last 2 weeks	88 (12.17)	26 (5.03)
Fever in last 2 weeks	234 (32.37)	87 (16.83)
Cough in last 2 weeks	418 (57.81)	188 (36.36)
Health status summary scores*		
Physical health summary score	43.74 (12.68)	45.02 (10.61)
Psychosocial health summary score	53.72 (7.28)	51.91 (8.53)

Data are mean (SD) or n (%). Age was calculated as of the interview date except in the analysis of health care utilization when age was calculated as of December 31, 2009.

*Health status questions were only applied to children ages 5 and above. Therefore, the youngest subgroup of children for these outcomes only contains children between the ages of 5-7 years.

### Health care utilization

**Figure 1:**
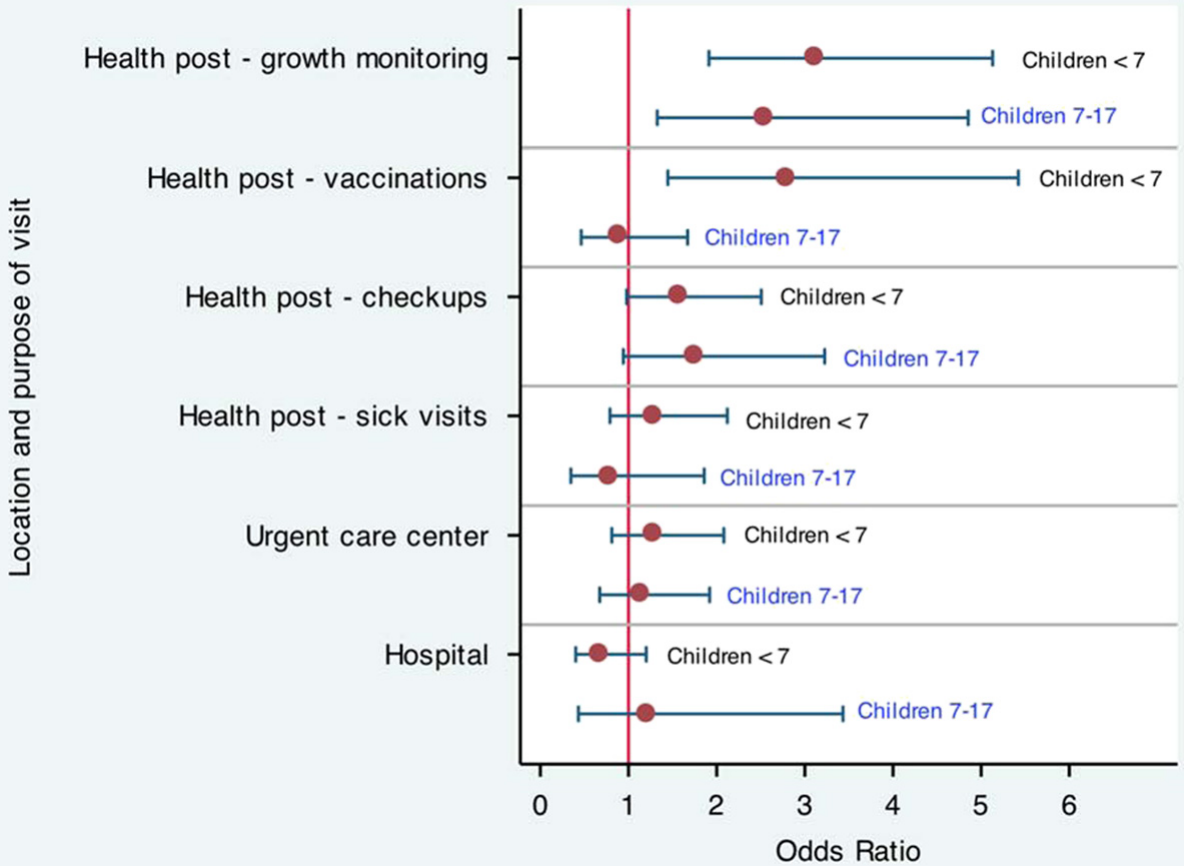
Odds ratios and 95% confidence intervals for the impact of Bolsa Família on health care utilization.

The analysis of health care utilization was restricted to children born before 2009 (1,119 children; 88% of whole sample), as the survey questions referred to the year 2009. For children under the age of seven years in 2009 (n = 629), Bolsa Família had a significant impact on several measures of health care utilization (Figure 1; Additional file 1: Table S1). Bolsa Família increased the odds of any health post visits for growth monitoring (odds ratio (OR) = 3.1; p < 0.001), vaccinations (OR = 2.8; p = 0.002), and checkups (OR = 1.6; p = 0.061). For older children between the ages of 7-17 years (n = 489), Bolsa Família increased the odds of any health post visits for growth monitoring (OR = 2.5; p = 0.005) and checkups (OR = 1.7; p = 0.077).

Bolsa Família also increased the number of health post visits for children under the age of seven years (Additional file 1: Table S2). Participation in the program increased the number of visits for growth monitoring and checkups in 2009 by 0.6 and 0.2 visits, respectively (p = 0.049 and p = 0.068, respectively). Bolsa Família was not associated with a significant change in the number of health post visits for older children between the ages of 7-17 years. The study was unable to detect a significant impact of Bolsa Família on visits to the health post when sick, the urgent care center, or the hospital.

### Illnesses

**Figure 2:**
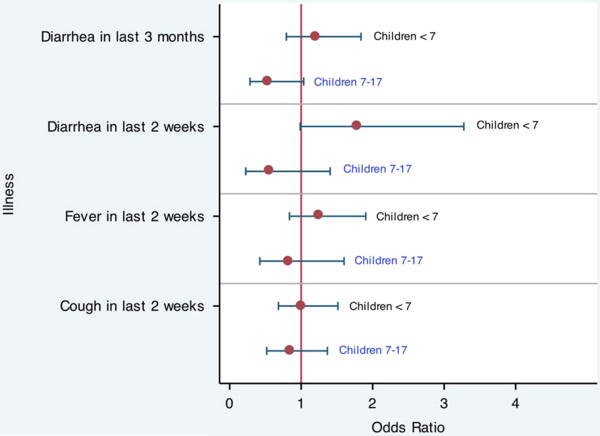
Odds ratios and 95% confidence intervals for the impact of Bolsa Família on illnesses.

The analysis of illness rates was restricted to children with the age of three months and older since respondents were asked about illnesses in the last three months and two weeks. Bolsa Família was associated with increased odds of having diarrhea in the last two weeks for younger children under seven (OR = 1.8; p = 0.055) and decreased odds of having diarrhea in the last three months for older children 7-17 (OR = 0.543; p = 0.064). These estimates may lack precision due to the small sample size and infrequent occurrences of diarrhea (only 16% of the older children 7-17 had diarrhea in the last three months; 12% of the younger children under the age of seven years had diarrhea in the last two weeks). Bolsa Família did not have a significant impact on odds of cough or fever in the last two weeks (Figure 2; Additional file 1: Table S3).

### Health status

The analysis of subjective health status, as reported by the mother, was limited to children with the age of five years and older, as the survey questions were developed for that age group. Bolsa Família did not have a statistically significant impact on physical health or psychosocial health summary scores for younger children between the ages of 5-7 years, but this may be due to the small sample size (n = 246). Among older children between the ages of 7-17 years (n = 517), who are not required to meet the health conditionalities, there were no significant differences in physical health summary scores between beneficiaries and non-beneficiaries, but beneficiaries had significantly better psychosocial health summary scores (β = 2.6; p = 0.007) (Table 4). This impact on psychosocial health is primarily attributable to Bolsa Família’s positive association with SF-10 questions related to children’s satisfaction with friendships and on their behavior compared to other children of the same age (individual survey items are shown in Additional file 1: Table S4).

**Table 4:** Impact of Bolsa Família on health status summary scores

	**Children 5-7 years**		**Children 7-17 years**	
	**β (95% CI)**	**p value**	**β (95% CI)**	**p value**
Physical health summary score	0.430 (-3.147, 4.007)	0.813	0.524 (-2.038, 3.087)	0.687
Psychosocial health summary score	1.924 (-0.5472, 4.394)	0.126	2.648 (0.720, 4.575)	0.007***

Coefficients of Bolsa Família treatment (β) were estimated while controlling for individual child characteristics (age, gender, race), mother’s characteristics (age, race, literacy, education), and household characteristics (water meter, light meter, closed sewer, total household income, number of household members, other government benefit, employment status of household members). Separate regressions were conducted—one for each outcome and age-group combination. Standard errors are robust and clustered at the household level. *p < 0.10, **p < 0.05, ***p < 0.01.

## Discussion

This community-based study finds that participation in Bolsa Família is associated with significantly better health outcomes along many different measures. These positive outcomes include greater utilization of preventive health care services and improved psychosocial health. Beneficiary children under the age of seven years are required to comply with the program’s health conditionalities, which include vaccinations and growth monitoring twice a year, and this study’s findings suggest that they are, in fact, complying with the health conditionalities. Beneficiary children are also more likely to obtain checkups, which is promising given that checkups are not required by the conditionalities.

These findings are consistent with recent studies that have reported an important positive impact of Bolsa Família on childhood mortality [23,24] and provide insights into how the program may improve child health. The implementation of Bolsa Família has been found to be associated with reductions in mortality in children younger than five years of age [23,24]. Furthermore, the program has had the greatest impact on mortality attributable to poverty-related causes [23] and sensitive to primary care services [24]. This study found that beneficiary children are, in fact, more likely to obtain primary care services. Future research should explore which program components have the greatest impact on health outcomes and, more specifically, whether reductions in child and infant mortality are primarily due to reductions in poverty from cash payments or increased utilization of health care services from the health conditionalities, or a combination of the program components.

Other CCT programs have been found to increase health care utilization. In Mexico, there was an increase of about two daily outpatient visits to health facilities in areas where Oportunidades was offered [3]. In Honduras, Programa de Asignación Familiar increased routine well-child checkups and growth monitoring visits for children by 19 and 15 percentage points, respectively [34]. Nicaragua’s Red de Proteccion Social increased the proportion of infants (0-3 years old) taken to health centers in the last six months by 19.5 percentage points after one year and 11 percentage points after two years [5].

In this study, positive spillover effects were observed for older siblings between the ages of 7-17 years who were no longer required to comply with the health conditionalities. Older siblings had increased odds of visiting the health post for growth monitoring and checkups. This may occur for several reasons. Mothers may take all of their children to the health post at the same time, or they may better understand the importance of preventive health care due to Bolsa Família. Alternatively, mothers may misunderstand the age range covered by the health conditionalities. Future research should examine why older siblings are utilizing more preventive health care though not required to.

That Bolsa Família has had an impact on children beyond the age range covered by the health conditionalities is a significant finding. To date, no studies have evaluated the health impacts of CCT programs on members of beneficiary families who are not directly targeted by the health conditonalities. Oportunidades was found to impact adult health, but its health conditionalities apply to the entire family. This study’s findings suggest that Bolsa Família may have important externalities. Program administrators should seize this opportunity to reach older children and other family members who may not be enrolled in Bolsa Família. The program’s lack of impact on vaccination visits for older children is not surprising, as most of the childhood vaccinations are administered through the age of six years [9].

Participation in Bolsa Família is also associated with a significantly better summary score for children’s psychosocial health. Bolsa Família is linked to improved satisfaction with friendships and better age-appropriate behavior. Mexico’s Oportunidades program was found to decrease aggressive and oppositional symptoms [35], perhaps due to the program’s impact on nutrition [36]. Supplementing the income of poor families can improve children’s behavior [37].

Bolsa Família did not have a significant impact on children’s health care utilization for illnesses or emergencies, illness rates, or physical health. Other studies have found mixed evidence of the impact of CCT programs on objective health measures, with positive impacts only among select sub-groups [8,38]. There are a few potential explanations. A longer time period may be needed for these health benefits to accrue and become apparent. CCT programs may be less effective at improving outcomes like diarrhea or physical functioning, as they may be more sensitive to interventions like improved sanitation and better built environments. Also, in this study site, the process for meeting the health conditionalities at the health post took place independently of other services, such as routine physical exams, which could potentially have a greater impact on health but which require appointments scheduled far in advance.

Bolsa Família encourages access to services at the health post but the quality and benefits of these services is unknown. For example, based on the observations of one author (AS), there was little, if any, follow-up to children’s growth monitoring, even for at-risk children, at the health post serving the study site. The benefits of improved access may be limited by the quality of existing services. The Family Health Program, which provides sends health workers into communities to deliver primary health care, may complement Bolsa Família and help families achieve greater health gains. Recent studies have reported on the important positive interaction between Bolsa Família and the Family Health Program [24,39].

This study has several limitations. The study focused on a slum community in northeastern Brazil and may not be generalizable to rest of the country, especially given likely regional variations. Access to health and education services may differ in rural areas. For example, Bolsa Família beneficiaries in this study had access to a local health post that was in close geographic proximity, but beneficiaries living in other areas, especially rural areas, may have limited access to health care services. Limited access to health care services may hinder families’ ability to enroll in Bolsa Família, meet the health conditionalities, and realize the program’s potential health gains. Despite the study’s focus on a slum community in a large urban center in the Northeast, its findings have important implications for the program, as both poverty and Bolsa Família payments and beneficiaries are disproportionately concentrated in the northeast. Furthermore, rapid urbanization and the concentration of poverty in major cities make slum communities extremely relevant to policy makers.

In addition, because the survey was administered in 2010, the sample was restricted to children at least three months old for questions related to illness rates, children at least five years old for questions related to health status, and children born before 2009 for questions related to health care utilization. The impact of Bolsa Família on children born more recently may differ from the impact found in this study, as the program may have enrolled different types of families in the last few years and access to and quality of health care services may also have changed. Future research should examine whether this study’s findings hold for more recent cohorts of patients.

Propensity score methods are based on an important assumption that outcomes are independent of program participation conditional on a set of observable characteristics and that there are no systematic differences in unobserved characteristics (e.g., motivation, risk aversion) between beneficiary and non-beneficiary groups which in turn may create selection bias. Typical unobserved characteristics relevant for social programs include motivation and connection to supportive social networks. This study attempted to address this limitation by capturing proxies for motivation (e.g., whether the household applied for government programs including Bolsa Família), social networks (e.g., whether the respondent knew any Bolsa Família beneficiaries, whether respondent belonged to a group), and other typically unobserved characteristics in the detailed questionnaire. However, any residual differences between the beneficiary and non-beneficiary groups could bias the results.

By examining program impacts on children of different ages, some of whom must comply with the health conditionalities and some of whom do not, this study’s findings indicate that different program components affect different outcomes. The health conditionalities likely have an impact on utilization of preventive health care services, and this impact spills over onto older siblings. The cash component provides a regular source of income which may impact psychosocial health. Future studies should further attempt to disentangle the relative importance of different program components.

## Conclusions

CCT programs like Bolsa Família have substantial potential to complement existing health care services and improve access to health care. This study finds that Bolsa Família increases utilization of preventive health care services. The program encourages poor families to use existing health services and to interact with the public health system, thereby providing an excellent opportunity to connect families with other services that may benefit them. By linking the health conditionalites to effective but underutilized services and improving the quality of existing health care services, programs like Bolsa Família may be able to further improve health outcomes and have a greater impact on the lives of poor families.

## Competing interests

We declare that we have no competing interests.

## Authors’ contributions

AS, FC, MGR, and AIK oversaw the design of the study. AS collected, analyzed, and interpreted the data and prepared the report. All authors approved the final version of the report.

## Pre-publication history

The pre-publication history for this paper can be accessed here:

http://www.biomedcentral.com/1472-698X/14/10/prepub

## Supplementary Material

Additional file 1: Figure S1 Flow diagram of study design. **Note A1.** Details of propensity score weighting method [40]. **Table S1.** Impact of Bolsa Família on health care utilization. **Table S2.** Impact of Bolsa Família on the amount of health care utilization. **Table S3.** Impact of Bolsa Família on illnesses. **Table S4.** Individual health status questions/items. Click here for file
