# Supplementary material for: The impact of Brazil’s Bolsa Família conditional cash transfer program on children’s health care utilization and health outcomes
Source: BMC Int Health Hum Rights. 2014 Apr 1;14:10. doi: 10.1186/1472-698X-14-10 (PMC4021270; doi:10.1186/1472-698X-14-10)
Supplement: Additional file 1: Figure S1 — Flow diagram of study design. Note A1. Details of propensity score weighting method [40]. Table S1. Impact of Bolsa Família on health care utilization. Table S2. Impact of Bolsa Família on the amount of health care utilization. Table S3. Impact of Bolsa Família on illnesses. Table S4. Individual health status questions/items. [file 1472-698X-14-10-S1.doc]

# Additional file 1

### Figure S1 – Flow diagram of study design

###

## Note A1 – Details of propensity score weighting method

If *p* is the predicted probability of being a Bolsa Família beneficiary, the weight would be 1-*p* for beneficiary children and *p* for non-beneficiary children. This technique has been used previously and avoids critiques of other propensity score adjustment approaches, such as the exclusion of data from the unmatched population which may occur with the matching approach and the potential for very high weights for individuals at the extreme values of propensity scores with the inverse probability weighting approach [40].

### Table S1 – Impact of Bolsa Família on health care utilization

|  | Children < 7 years | | Children 7-17 years | |
| --- | --- | --- | --- | --- |
|  | OR (95% CI) | p value | OR (95% CI) | p value |
| Any health post visit – growth monitoring | 3.132  (1.912, 5.130) | 5.75e-06*** | 2.537  (1.326, 4.853) | 0.005*** |
| Any health post visit – vaccination | 2.800  (1.446, 5.422) | 0.002*** | 0.880  (0.463, 1.670) | 0.695 |
| Any checkup | 1.566  (0.979, 2.506) | 0.061* | 1.743  (0.942, 3.225) | 0.077* |
| Any health post visit – sick | 1.294  (0.790, 2.121) | 0.306 | 0.803  (0.347, 1.859) | 0.608 |
| Any urgent care center visit | 1.299  (0.811, 2.081) | 0.276 | 1.137  (0.673, 1.920) | 0.631 |
| Any hospital visit | 0.694  (0.402, 1.201) | 0.192 | 1.218  (0.432, 3.433) | 0.710 |

Odds ratios (OR) were estimated while controlling for individual child characteristics (age, gender, race), mother’s characteristics (age, race, literacy, education), and household characteristics (water meter, light meter, closed sewer, total household income, number of household members, other government benefit). Separate logistic regressions were conducted—one for each outcome and age-group combination. Standard errors are robust and clustered at the household level. * p<0.10, ** p<0.05, *** p<0.01

## Table S2 – Impact of Bolsa Família on the amount of health care utilization

|  | Children < 7 years | | Children 7-17 years | |
| --- | --- | --- | --- | --- |
|  | β (95% CI) | p value | β (95% CI) | p value |
| Health post visits – growth monitoring | 0.621  (0.002, 1.239) | 0.049** | 0.120  (-0.344, 0.585) | 0.611 |
| Health post visits – vaccination | 0.212  (-0.046, 0.470) | 0.108 | -0.082  (-0.266, 0.102) | 0.381 |
| Checkups | 0.232  (-0.017, 0.482) | 0.068* | 0.087  (-0.171, 0.344) | 0.509 |
| Health post visits – sick | 0.142  (-0.114, 0.397) | 0.276 | -0.004  (-0.186, 0.178) | 0.967 |
| Urgent care center visits | 0.163  (-0.278, 0.604) | 0.468 | -0.107  (-0.494, 0.279) | 0.586 |
| Hospital visits | -0.163  (-0.333, 0.006) | 0.059* | -0.020  (-0.173, 0.133) | 0.797 |

Coefficients of Bolsa Família treatment (β) were estimated while controlling for individual child characteristics (age, gender, race), mother’s characteristics (age, race, literacy, education), and household characteristics (water meter, light meter, closed sewer, total household income, number of household members, other government benefit). Separate linear regressions were conducted—one for each outcome and age-group combination. Standard errors are robust and clustered at the household level. * p<0.10, ** p<0.05, *** p<0.01

## Table S3 – Impact of Bolsa Família on illnesses

|  | Children < 7 years | | Children 7-17 years | |
| --- | --- | --- | --- | --- |
|  | OR (95% CI) | p value | OR (95% CI) | p value |
| Diarrhea in last 3 months | 1.207  (0.793, 1.837) | 0.380 | 0.543  (0.285, 1.035) | 0.064* |
| Diarrhea in last 2 weeks | 1.798  (0.987, 3.275) | 0.055* | 0.562  (0.224, 1.405) | 0.218 |
| Fever in last 2 weeks | 1.262  (0.837, 1.903) | 0.267 | 0.822  (0.422, 1.600) | 0.563 |
| Cough in last 2 weeks | 1.016  (0.682, 1.514) | 0.937 | 0.842  (0.519, 1.367) | 0.487 |

Odds ratios (OR) were estimated while controlling for individual child characteristics (age, gender, race), mother’s characteristics (age, race, literacy, education), and household characteristics (water meter, light meter, closed sewer, total household income, number of household members, other government benefit). Separate logistic regressions were conducted—one for each outcome and age-group combination. Standard errors are robust and clustered at the household level. Children are at least 3 months old. * p<0.10, ** p<0.05, *** p<0.01

## Table S4 – Individual health status questions/items

|  | Children 5-7 years | | Children 7-17 years | |
| --- | --- | --- | --- | --- |
|  | β (95% CI) | p value | β (95% CI) | p value |
| Item 1  (health in general) | 0.324  (-0.099, 0.746) | 0.131 | 0.0467  (-0.252, 0.345) | 0.759 |
| Item 2a  (limitations to activities requiring energy due to health problems) | 0.001  (-0.423, 0.424) | 0.998 | -0.0384  (-0.285, 0.208) | 0.760 |
| Item 2b  (limitations to activities requiring bending, lifting, or stooping due to health problems) | 0.002  (-0.242, 0.245) | 0.989 | 0.221  (-0.022, 0.465) | 0.074* |
| Item 3  (limitations to schoolwork or activities due to physical health problems) | -0.306  (-0.748, 0.136) | 0.173 | -0.089  (-0.364, 0.185) | 0.522 |
| Item 4  (limitations to schoolwork or activities due to emotional or behavioral problems) | 0.063  (-0.163, 0.289) | 0.582 | 0.103  (-0.113, 0.319) | 0.349 |
| Item 5  (bodily pain or discomfort) | 0.137  (-0.274, 0.548) | 0.511 | 0.0521  (-0.323, 0.427) | 0.785 |
| Item 6  (satisfaction with friendships) | 0.140  (-0.142, 0.422) | 0.328 | 0.381  (0.073, 0.690) | 0.016** |
| Item 7  (satisfaction with life overall) | 0.371  (0.005, 0.737) | 0.047** | 0.234  (-0.100, 0.569) | 0.169 |
| Item 8  (time acting bothered or upset) | 0.376  (-0.099, 0.851) | 0.120 | 0.145  (-0.193, 0.483) | 0.398 |
| Item 9  (behavior in general) | 0.083  (-0.443, 0.608) | 0.757 | 0.483  (0.151, 0.815) | 0.004*** |

Coefficients of Bolsa Família treatment (β) were estimated while controlling for individual child characteristics (age, gender, race), mother’s characteristics (age, race, literacy, education), and household characteristics (water meter, light meter, closed sewer, total household income, number of household members, other government benefit). Separate linear regressions were conducted—one for each outcome and age-group combination. Standard errors are robust and clustered at the household level. * p<0.10, ** p<0.05, *** p<0.01
